# Increased Vitamin D Receptor Immunoreactivity in Endometrial Polyps: An Exploratory Single-Center Pilot Study

**DOI:** 10.7759/cureus.98863

**Published:** 2025-12-10

**Authors:** Virginia Araujo Andrade, Marcelo Antonini, Rafael de Deus Moura, Pedro Vitor Lopes Costa

**Affiliations:** 1 Obstetrics and Gynaecology, Federal University of Piauí, Teresina, BRA; 2 Mastology, State Public Servant Hospital - Francisco Morato de Oliveira, Sao Paulo, BRA; 3 Pathology, Federal University of Piauí, Teresina, BRA; 4 Gynaecology, Hospital Getúlio Vargas, Teresina, BRA

**Keywords:** endometrial hyperplasia, endometrial polyps, immunohistochemistry staining, translational medicine research, vitamin-d receptor

## Abstract

Objective: This study aimed to compare vitamin D receptor (VDR) immunoexpression in endometrial polyps and paired adjacent normal endometrium.

Methods: Seventeen women (mean age, 48 ± 9 years) undergoing hysteroscopic polypectomy were included. Paired samples of polyp tissue and adjacent normal endometrium were analyzed by standardized immunohistochemistry using an anti-VDR monoclonal antibody. Two blinded pathologists independently assessed staining intensity (0-3) and fraction of positive cells (0-2), and calculated a composite van Slooten score (0-6). VDR positivity was defined as a composite score ≥ 3.

Results: VDR expression was higher in polyps than in normal endometrium. Mean intensity (1.5 ± 1.1 vs. 0.8 ± 0.8; p = 0.041), fraction of stained cells (1.1 ± 0.7 vs. 0.4 ± 0.7; p = 0.020), and composite score (2.6 ± 2.0 vs. 1.0 ± 1.5; p = 0.020) were significantly greater in polyps. VDR positivity was observed in 58.8% (10/17) of polyps and 23.5% (4/17) of normal endometrial samples (p = 0.037). Interobserver agreement was substantial to almost perfect (κ = 0.70-0.85). No association was observed between VDR score and age, BMI, or menopausal status.

Conclusions: Endometrial polyps exhibit increased VDR expression compared with paired normal endometrium. These exploratory findings suggest compensatory VDR upregulation in benign proliferative lesions, warranting confirmation in larger molecular studies.

## Introduction

The vitamin D receptor (VDR) has emerged as a critical mediator of cellular homeostasis beyond its classical role in calcium metabolism, with mounting evidence supporting its involvement in antiproliferative, proapoptotic, and anti-inflammatory pathways across diverse tissues [1,2]. The nuclear receptor functions as a ligand-activated transcription factor that, upon binding 1,25-dihydroxyvitamin D3 (calcitriol), heterodimerizes with retinoid X receptor and modulates gene expression through vitamin D response elements [3]. This regulatory network influences cell cycle progression, apoptosis, angiogenesis, and immune responses - processes fundamentally altered in neoplastic transformation [4].

Epidemiological studies consistently demonstrate inverse associations between vitamin D status and cancer incidence across multiple organ systems, including colorectal, prostate, and breast malignancies [5-7]. Importantly, increased VDR expression has been documented in various benign proliferative lesions, including colorectal adenomas and nasal polyps, suggesting upregulation may represent an intrinsic protective mechanism against uncontrolled growth [8,9]. This pattern implies that tissues experiencing hyperplastic stimuli may increase VDR expression as an adaptive response to excessive proliferative stimulation mediated by estrogenic activity, inflammatory mediators, and growth-related pathways, aiming to enhance vitamin D signaling in order to limit cell proliferation, promote apoptosis, and restore tissue homeostasis.

Within gynecological pathology, vitamin D signaling demonstrates tissue-specific effects. In endometriosis, vitamin D exhibits anti-inflammatory and antiproliferative properties [10,11], while uterine leiomyomas show reduced VDR expression compared to normal myometrium [12]. These findings suggest that VDR expression patterns may reflect tissue-specific responses to pathological stimuli and influence disease progression.

Endometrial polyps represent focal hyperplastic overgrowths of endometrial glands and stroma surrounding a central vascular core, affecting 6-32% of women depending on population and diagnostic methodology [13,14]. While typically benign, a subset harbors premalignant or malignant changes, particularly in postmenopausal women with bleeding or those receiving tamoxifen therapy [15]. The pathogenesis involves complex interactions between hormonal imbalances (especially unopposed estrogen), altered steroid receptor expression, chronic inflammation, and dysregulated apoptotic signaling [16,17]. Recent molecular analyses have identified oncogenic mutations (K-RAS) and transcription factor rearrangements (HMGA1/2), further complicating the pathophysiological landscape [18,19].

Given vitamin D's established roles in modulating estrogen receptor expression, suppressing inflammatory cytokines, and promoting apoptosis - processes central to endometrial polyp pathogenesis - investigating VDR expression in these lesions represents a logical translational approach. However, no studies have directly examined VDR presence or functional significance in endometrial polyps, representing a significant knowledge gap given the documented protective role of vitamin D signaling in analogous hyperplastic conditions [20,21].

The identification of reliable biomarkers for benign endometrial proliferative disorders remains a critical research need. The present study was designed as an exploratory pilot investigation and is not intended to establish biomarker validity. Instead, we sought to generate preliminary data on VDR expression in endometrial polyps to guide future, adequately powered studies. Understanding the molecular mechanisms underlying differential VDR expression could inform the development of targeted interventions and improve patient stratification strategies.

The identification of reliable molecular markers for benign endometrial proliferative disorders remains an important unmet need. Understanding VDR expression patterns in endometrial polyps may provide mechanistic insights into polyp pathophysiology and, in future studies, help to identify candidate tissue biomarkers for risk stratification. Although these clinical applications remain hypothetical, they may inform investigations focusing on therapeutic strategies targeting the vitamin D pathway, particularly in patients with recurrent polyps, metabolic risk factors, or surgical contraindications. In this study, we hypothesized that endometrial polyps would demonstrate increased VDR expression compared with adjacent normal endometrium, consistent with compensatory upregulation in response to hyperplastic stimuli.

## Materials and methods

Study design and participants

This cross-sectional study was conducted at the University Hospital of the Federal University of Piauí (HU-UFPI), Teresina, Brazil, and included women who underwent hysteroscopic polypectomy between May and June 2022. The inclusion criteria required histologically confirmed endometrial polyps with available adjacent normal endometrial tissue. Patients who had received hormonal treatments (including hormonal contraceptives or hormone replacement therapy) within the three months preceding surgery were excluded to minimize potential hormonal influence on VDR expression. The menstrual phase of the endometrium was not systematically recorded, which we acknowledge as a limitation when interpreting the results. Clinical data collected included age, menopausal status, BMI, comorbidities, and presenting symptoms. Of the 24 women initially recruited, seven were excluded: three due to recent use of hormonal therapy within the previous three months and four for lack of adequate paired normal endometrial tissue. The final analytical cohort, therefore, comprised 17 patients. This investigation was conceived as an exploratory, single-center pilot study based on a convenience sample of consecutive eligible women recruited over a predefined period. No formal a priori sample size calculation or power analysis was performed, as the primary aim was to generate preliminary data on VDR expression in endometrial polyps to inform the design of future, adequately powered studies.

Specimen collection and processing

During hysteroscopic procedures, paired tissue samples were collected: one from the endometrial polyp and one from morphologically normal adjacent endometrium. All specimens underwent immediate fixation in 10% neutral buffered formalin, paraffin embedding, and sectioning at 4-μm thickness for histopathological and immunohistochemical (IHC) analysis [22,23].

Histopathological evaluation

Conventional hematoxylin-eosin staining confirmed histological diagnoses according to World Health Organization criteria [24]. Only samples definitively classified as benign endometrial polyps and normal endometrial tissue were retained for IHC evaluation. Histological classification of the polyps was performed according to World Health Organization criteria, distinguishing between functional and hyperplastic types

IHC analysis

VDR immunostaining was performed at a certified pathology laboratory (APC, São Paulo, Brazil) using the standardized EnVision FLEX High pH system (Dako®, Agilent Technologies, Santa Clara, California, USA). Following deparaffinization and rehydration, antigen retrieval was employed using a high-pH buffer in a PT-Link® system (Dako®) (97°C, 20 minutes). Sections were incubated with anti-VDR antibody (Biogen®, Cambridge, Massachusetts, USA; clone D6, mouse monoclonal IgG2a/κ) for 60 minutes at 37°C. Detection utilized 3,3′-diaminobenzidine (DAB) chromogen with Harris hematoxylin counterstaining. Renal tubule tissue served as a positive control, with negative controls omitting the primary antibody [25,26].

Quantitative assessment

Two experienced pathologists, blinded to sample identity, independently evaluated all slides. VDR expression was quantified using the semi-quantitative method proposed by van Slooten et al. [26], which combines staining intensity (0 (absent), 1 (weak), 2 (moderate), 3 (strong)) and the fraction of positively stained epithelial cells (0 (0-25%), 1 (26-75%), 2 (76-100%)). A composite IHC score ranging from 0 to 6 was calculated based on the combined assessment of these parameters. Cases with scores ≥ 3 were classified as VDR-positive, as previously described in the literature.

**Figure 1:**
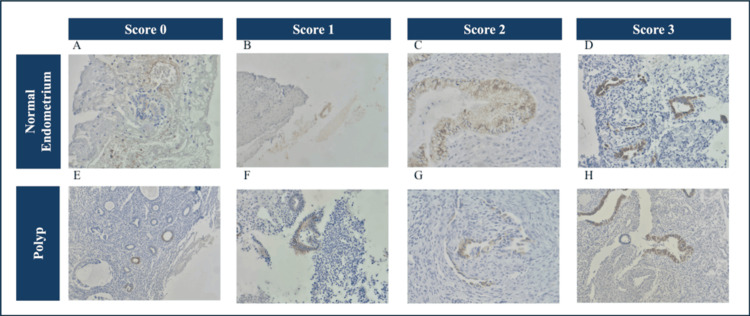
Immunoexpression of vitamin D receptor (VDR) in normal endometrium and endometrial polyps. Representative immunohistochemical staining patterns of VDR expression in normal endometrial tissue (A-D) and endometrial polyps (E-H). Images show composite scores from 0 to 3: (A, E) Score 0: no detectable staining; (B, F) Score 1: weak and/or focal staining; (C, G) Score 2: moderate and more diffuse staining; (D, H) Score 3: strong staining in the majority of epithelial cells. Magnification: 400×. All images were obtained from original material processed and analyzed by the authors.

Representative IHC staining patterns used to illustrate each scoring category are shown in Figure 1. These micrographs display increasing staining intensity from absent to strong nuclear reactivity in both normal endometrial tissue and endometrial polyps.

The individual case-level results obtained from both observers, including staining intensity, fraction of positive epithelial cells, composite score, and concordance status, are detailed in Table 1. This table lists all 17 paired samples (polyp and adjacent normal endometrium) analyzed.

**Table 1:** Individual case-level immunohistochemical scores for VDR expression in endometrial polyps and adjacent normal endometrium. VDR: vitamin D receptor

Patient Number	Tissue Slide	Staining Intensity		Fraction Cells		Score		Concordance
		Observer 1 (%)	Observer 2 (%)	Observer 1 (%)	Observer 2 (%)	Observer 1 (%)	Observer 2 (%)	
1	Polyp	2	2	80	80	4	4	Yes
	Normal	2	2	90	90	4	4	Yes
2	Polyp	2	2	70	70	3	3	Yes
	Normal	1	1	70	70	2	2	Yes
3	Polyp	3	3	80	80	6	6	Yes
	Normal	2	2	80	80	4	4	Yes
4	Polyp	2	2	65	65	3	4	No
	Normal	1	1	90	90	4	4	Yes
5	Polyp	2	2	10	10	3	3	Yes
	Normal	3	3	10	10	2	2	Yes
6	Polyp	0	0	90	90	0	1	No
	Normal	0	0	90	90	5	5	Yes
7	Polyp	0	0	0	0	0	0	Yes
	Normal	2	2	0	0	0	0	Yes
8	Polyp	2	2	0	0	3	3	Yes
	Normal	2	2	20	20	2	2	Yes
9	Polyp	2	2	80	80	4	4	Yes
	Normal	2	2	50	50	3	3	Yes
10	Polyp	1	1	90	90	2	2	Yes
	Normal	1	1	100	100	4	4	Yes
11	Polyp	1	1	80	80	2	2	Yes
	Normal	2	2	80	80	3	3	Yes
12	Polyp	1	1	10	10	1	1	Yes
	Normal	2	2	80	80	4	4	Yes
13	Polyp	1	1	15	15	1	1	Yes
	Normal	2	2	10	10	2	2	Yes
14	Polyp	2	2	20	20	3	3	Yes
	Normal	2	2	50	50	3	3	Yes
15	Polyp	1	1	60	60	1	1	Yes
	Normal	0	0	0	0	0	1	No
16	Polyp	2	2	65	65	3	3	Yes
	Normal	1	1	10	10	1	1	Yes
17	Polyp	2	2	70	70	3	4	No
	Normal	0	0	0	0	0	0	Yes

**Figure 2:**
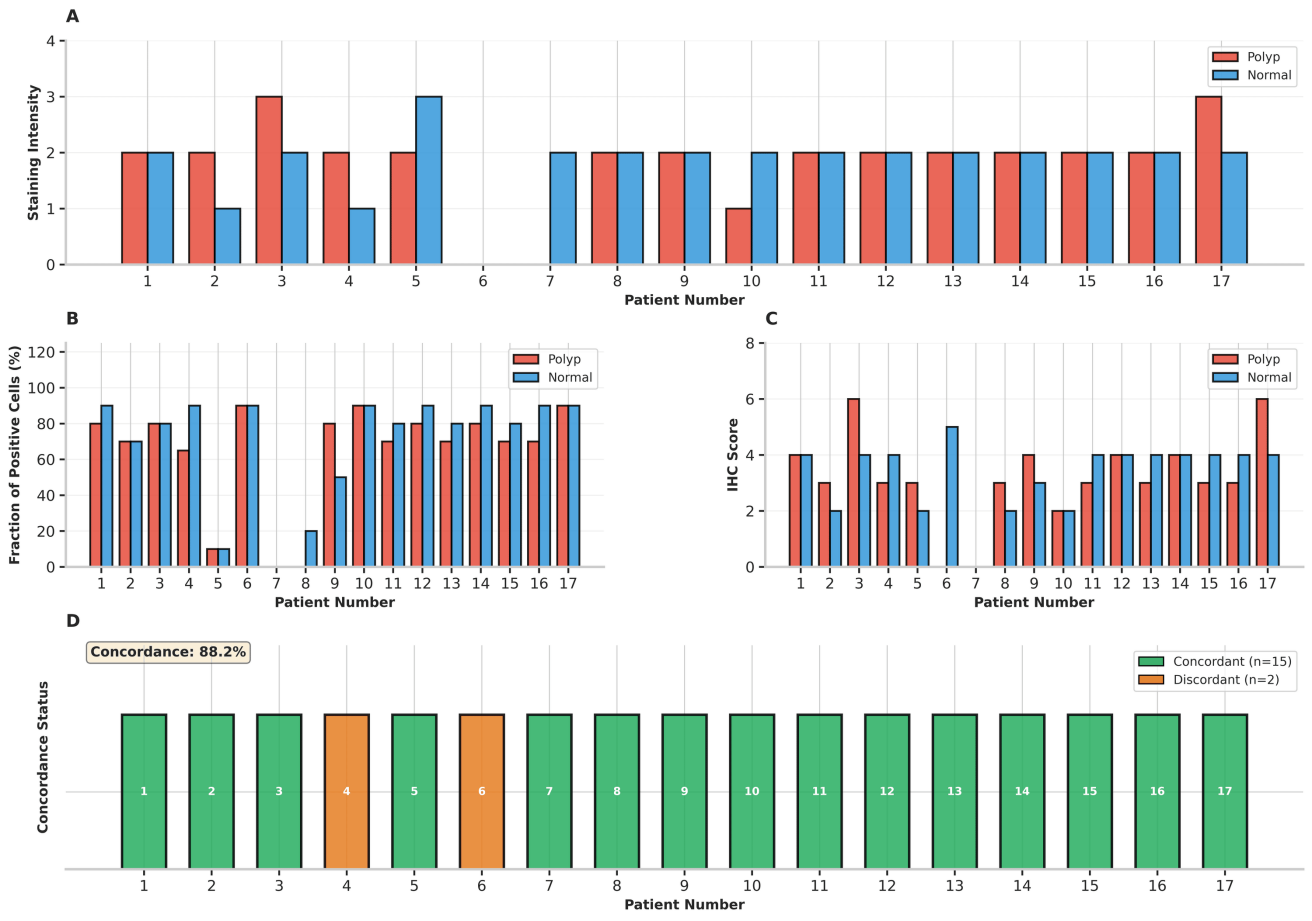
Quantitative assessment and visualization of VDR expression patterns Quantitative and interobserver analysis of vitamin D receptor (VDR) immunohistochemistry. (A) Staining intensity scores, (B) fraction of positively stained epithelial cells, (C) composite immunohistochemical (IHC) scores, and (D) interobserver concordance across 17 paired samples of endometrial polyps and adjacent normal endometrium. The overall concordance rate was 88.2%, with κ = 0.77 for polyp tissue and κ = 0.85 for normal endometrium, confirming high scoring reproducibility.

The overall distribution of staining intensity, fraction of stained cells, composite IHC scores, and interobserver agreement for the entire cohort is summarized graphically in Figure 2. This figure provides a quantitative visualization of the data set and supports the reproducibility of the semiquantitative scoring method applied.

Interobserver reproducibility was evaluated using Cohen’s kappa coefficient (κ) to determine agreement between the two pathologists. Concordance status for each case was recorded and expressed as an overall agreement percentage.

Statistical analysis

Data analysis employed IBM SPSS Statistics for Windows, Version 20 (Released 2011; IBM Corp., Armonk, New York, United States) and R Project version 3.6.0 (R Foundation for Statistical Computing, Vienna, Austria). Descriptive statistics included means ± standard deviations for continuous variables and frequencies for categorical variables. Distribution normality was assessed using Shapiro-Wilk tests. Between-group comparisons utilized Mann-Whitney U tests for continuous scores and chi-square or Fisher's exact tests for categorical variables. Statistical significance was defined as p<0.05, with 95% confidence intervals reported where appropriate.

Ethical approval

The study was approved by the Research Ethics Committee of the Federal University of Piauí under protocol CAAE 55483521.3.0000.5214 and complied with the Brazilian National Health Council Resolution 466/2012. All participants signed a written informed consent form before enrollment.

## Results

Patient characteristics

Of the 24 women initially recruited, 17 met the inclusion criteria (70.8 %) and comprised the final analytical cohort. The mean age was 50 ± 12.3 years (range 31-70), with seven (41.2 %) postmenopausal and 10 (58.8 %) premenopausal participants. Abnormal uterine bleeding was the main presenting symptom in 12 cases (70.6 %), and five (29.4 %) were incidental findings. Regarding BMI, three women (17.6 %) were normal weight (18.5-24.9 kg/m^2^), eight (47.1 %) were overweight (25-29.9 kg/m^2^), and six (35.3%) were obese (≥30 kg/m^2^). Comorbidities included arterial hypertension in five (29.4%), dyslipidemia in one (5.9%), hypothyroidism in one (5.9%), and no comorbidities in 10 (58.8%). These clinical and pathological characteristics are summarized in Table 2.

**Table 2:** Clinical and pathological characteristics. Values are presented as mean ± standard deviation (SD) or as frequency (percentage), as appropriate. BMI is expressed in kilograms per square meter (kg/m^2^).

Characteristics	n=17	%
Age (years)		
Mean, standard deviation	50 ± 12.3 anos	
Range (min-max)	21-70	
Menopausal status		
Postmenopausal	7	41.2
Premenopausal	10	58.8
Body mass index (BMI, kg/m^2^)		
Normal weight (18.5-24.9)	3	17.6
Overweight (25-29.9)	8	47.1
Obesity (≥30)	6	35.3
Comorbidities		
Arterial hypertension	5	29.5
Dyslipidemia	1	5.9
Hypothyroidism	1	5.9
None	10	58.8
Clinical symptoms		
Abnormal uterine bleeding	12	70.6
Incidental finding	5	29.7

VDR expression analysis and interobserver agreement

VDR expression was evaluated using the semiquantitative scoring system described in the Materials and Methods section. Normal endometrium predominantly exhibited weak or absent nuclear staining, whereas endometrial polyps showed moderate to strong nuclear immunoreactivity. Interobserver reproducibility between the two pathologists was substantial to almost perfect (κ = 0.70-0.85), confirming the reliability of the scoring system.

Comparative analysis

A comparative non-parametric analysis was performed to evaluate differences in VDR expression between endometrial polyps and paired normal endometrium. The results consistently demonstrated significantly higher VDR expression in polyps across all evaluated parameters.

Mean staining intensity was 1.5 ± 1.1 in polyps versus 0.8 ± 0.8 in normal endometrium (p = 0.041), and the mean fraction of positively stained epithelial cells was 1.1 ± 0.7 versus 0.4 ± 0.7 (p = 0.020). Consequently, the composite van Slooten score, which integrates intensity and fraction, was markedly higher in polyps (2.6 ± 2.0 vs 1.0 ± 1.5; p = 0.020).

Normality of data distribution was assessed using the Shapiro-Wilk test (W = 0.95, p = 0.36 for polyps; W = 0.94, p = 0.31 for normal endometrium), confirming the suitability of non-parametric testing. These quantitative results are presented in Tables 3-4.

**Table 3:** Composite van Slooten scores for VDR expression. Distribution of composite Van Slooten scores for vitamin D receptor (VDR) expression in endometrial polyps and normal endometrium. Data are presented as absolute frequencies and percentages. Statistical analysis used the Shapiro-Wilk test (W-value) for normality and the Mann-Whitney U test (U-value) for group comparison.

Composite Score	Polyp (n = 17)	Normal Endometrium (n = 17)	P-value
0	4 (23.5%)	10 (58.8%)	0.020
1	3 (17.6%)	3 (17.6%)	
2	0 (0.0%)	0 (0.0%)	
3	3 (17.6%)	2 (11.8%)	
4	4 (23.5%)	2 (11.8%)	
5	2 (11.8%)	0 (0.0%)	
6	1 (5.9%)	0 (0.0%)	
Mean ± SD	2.6 ± 2.0	1.0 ± 1.5	
Shapiro-Wilk for normality	W = 0.950, p = 0.36	W = 0.940, p = 0.31	-
Mann-Whitney (U)	U = 69.0, p = 0.020	-	-

**Table 4:** Staining intensity and fraction of positive cells for VDR expression. Distribution of staining intensity and fraction of positively stained cells for vitamin D receptor (VDR) expression in endometrial polyps and normal endometrium. Data are expressed as absolute numbers and percentages. Distribution normality was evaluated using the Shapiro-Wilk test (W-value), and intergroup comparisons were performed using the Mann-Whitney U test (U-value).

Score	Staining Intensity Polyp (n = 17)	Staining Intensity Normal Endometrium (n = 17)	P-value	Fraction of Positive Cells Polyp (n = 17)	Fraction of Positive Cells Normal Endometrium (n = 17)	P-value
0	4 (23.5%)	8 (47.1%)	0.041	4 (23.5%)	12 (70.6%)	0.020
1	3 (17.6%)	5 (29.4%)		8 (47.1%)	4 (23.5%)	
2	7 (41.2%)	4 (23.5%)		5 (29.4%)	1 (5.9%)	
3	3 (17.6%)	0 (0.0%)		-	-	
Mean ± SD	1.5 ± 1.1	0.8 ± 0.8		1.1 ± 0.7	0.4 ± 0.7	
Shapiro-Wilk (W)			W = 0.954, p = 0.39	Shapiro-Wilk (W)		W = 0.947, p = 0.35
Mann-Whitney (U)			U = 71.0, p = 0.041	Mann-Whitney (U)		U = 68.0, p = 0.020

**Figure 3:**
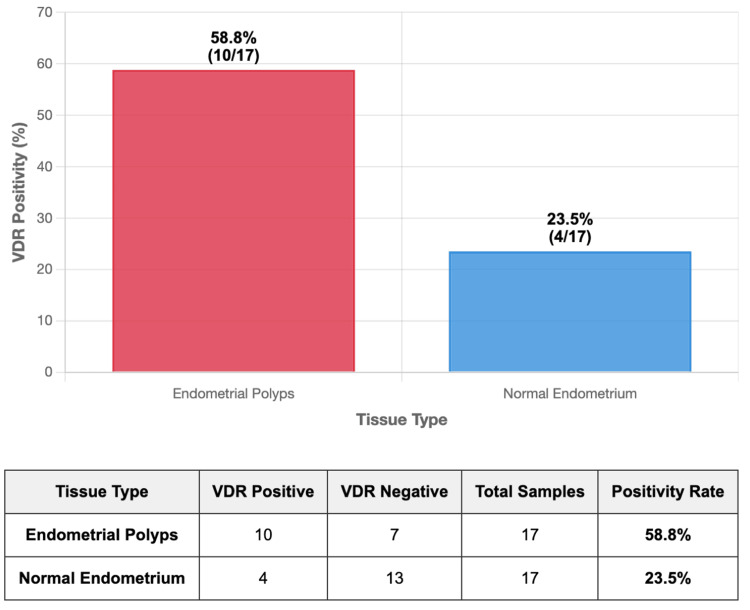
Comparative analysis of vitamin D receptor (VDR) expression between endometrial polyps and normal endometrium. Frequency of positive VDR expression in endometrial polyp versus adjacent normal endometrium. Percentage of tissue samples showing positive VDR expression (composite score ≥3) based on immunohistochemical analysis. VDR positivity was found to be significantly higher in polyp samples (58.8%, n = 10/17) compared to normal endometrial samples (23.5%, n = 4/17). The difference was found to be statistically significant (p = 0.037; chi-square test). The data represent paired tissue samples from 17 women undergoing hysteroscopic polypectomy.

Using the predefined threshold for VDR positivity (composite score ≥ 3), 58.8% (10/17) of polyp samples were classified as VDR-positive, compared with 23.5% (4/17) of normal endometrial samples (p = 0.037; χ^2^ test). This 2.5-fold increase reinforces the observation of enhanced receptor expression in benign hyperplastic lesions. The distribution of VDR-positive and negative cases is illustrated in Figure 3.

No statistically significant associations were found between VDR expression and age, menopausal status, BMI, or comorbidities (p > 0.05), suggesting that receptor upregulation in polyps is primarily a local phenomenon, independent of systemic metabolic or hormonal influences.

Overall, the results demonstrate a consistent and statistically significant increase in VDR immunoexpression in endometrial polyps compared with adjacent normal endometrium, supporting the hypothesis of receptor modulation associated with proliferative activity in benign endometrial lesions.

## Discussion

This study provides novel evidence of differential VDR expression in endometrial polyps, demonstrating significantly increased receptor levels compared to adjacent normal endometrium. These findings support our hypothesis that VDR upregulation represents a compensatory mechanism in benign hyperplastic endometrial lesions, potentially functioning to restore cellular homeostasis in response to dysregulated proliferative stimuli. Nevertheless, these findings must be interpreted with caution, given the study's limited sample size and the exclusive use of immunohistochemistry. Although the methodology was found to be standardized and yielded consistent interobserver results, the lack of complementary molecular or functional assays restricts our ability to draw definitive mechanistic conclusions.

These findings should be interpreted as exploratory and hypothesis-generating, given the small sample size and the methodological scope. The study was not designed or powered to establish VDR as a biomarker, but rather to provide preliminary observations that may inform future investigations. The observed 2.5-fold increase in VDR positivity rates (58.8% vs. 23.5%) represents a substantial biological difference that aligns with established patterns in other hyperplastic conditions. Recent studies have documented similar VDR overexpression in various benign proliferative lesions, supporting a conserved protective mechanism across tissues experiencing hyperplastic transformation [27-30]. Notably, VDR expression patterns appear to correlate with histological grade and differentiation status, as demonstrated in endometrioid carcinoma, where VDR displacement was found to be significantly associated with lower histological grade [31].

The mechanistic rationale for VDR upregulation in endometrial polyps likely involves the complex interplay between estrogen dominance and vitamin D signaling. Endometrial polyps typically develop in estrogen-rich environments, particularly during perimenopause when progesterone opposition diminishes [32]. Vitamin D has been shown to modulate estrogen receptor expression and activity, potentially serving as a regulatory counterbalance to unopposed estrogenic stimulation [33]. Recent evidence from endometrial hyperplasia studies demonstrates that vitamin D supplementation can improve metabolic profiles and clinical responses, supporting the therapeutic potential of vitamin D pathway modulation [34]. The increased VDR expression observed in our study may represent an adaptive response aimed at mitigating estrogen-driven hyperplasia through calcitriol-mediated growth inhibition. This concept is further supported by studies in uterine leiomyomas, where VDR expression was found to be significantly lower in neoplastic tissue compared to normal myometrium, suggesting that reduced VDR expression may facilitate aberrant growth [35].

Similar patterns of VDR upregulation have been documented in other benign hyperplastic conditions. Transcriptomic profiling of uterine leiomyomas has revealed increased VDR mRNA expression in specific tumor regions, suggesting localized adaptive responses within hyperplastic lesions [36]. Additionally, integrative genomic and transcriptomic analyses of fibroids have provided molecular signatures consistent with VDR pathway activation [37]. Beyond the gynecological field, VDR upregulation has also been described in colorectal adenomas and nasal polyps, supporting the notion that this receptor’s increased expression may represent a conserved feature across benign proliferative disorders.

Our findings require careful interpretation in light of recent contradictory evidence. Özakşit et al. (2016) reported no association between serum vitamin D levels and endometrial polyp formation (p = 0.583), concluding that vitamin D deficiency was not a risk factor for polyp development. However, this apparent contradiction may reflect fundamental differences between systemic vitamin D status and local tissue VDR expression. While serum vitamin D levels indicate systemic availability of the hormone, tissue-specific VDR expression represents the cellular capacity to respond to vitamin D signaling, which may be independently regulated [38,39]. This distinction is particularly relevant given recent advances in understanding local vitamin D metabolism within reproductive tissues. Studies have demonstrated that endometrial tissue expresses vitamin D metabolizing enzymes (1α-hydroxylase and 24-hydroxylase), enabling local conversion of circulating 25(OH)D3 to active calcitriol [40]. Therefore, elevated VDR expression in polyps may represent a compensatory upregulation in response to local vitamin D insufficiency or impaired signaling, independent of systemic vitamin D status.

Our results align with emerging evidence of VDR involvement in various gynecological pathologies. In endometriosis, VDR expression is upregulated in both eutopic and ectopic endometrium, and vitamin D deficiency has been identified as playing a significant role in disease pathogenesis [40]. Similarly, studies in polycystic ovary syndrome (PCOS) have revealed that hyperandrogenemia impairs endometrial VDR expression, suggesting that hormonal imbalances can directly affect vitamin D signaling pathways [40]. The pattern of VDR upregulation observed in endometrial polyps is consistent with protective responses documented in other benign hyperplastic conditions. In mammary tissue, VDR expression has been shown to correlate with differentiation status and may serve as a protective factor against malignant transformation [39]. This supports the hypothesis that increased VDR expression in polyps represents an intrinsic cellular defense mechanism against uncontrolled proliferation.

The differential VDR expression observed in endometrial polyps provides important insights for translational medicine applications. The 2.5-fold increase in VDR positivity suggests a tissue-specific response that could be exploited therapeutically. This upregulation may represent an endogenous protective mechanism that could be enhanced through targeted interventions [38]. From a biomarker perspective, VDR expression patterns could be further explored in future studies for potential use in polyp characterization and risk stratification. While intriguing, any therapeutic implications, including vitamin D-based interventions, remain hypothetical and require validation in larger, mechanistically oriented cohorts. Furthermore, the substantial interobserver agreement (κ = 0.70-0.85) supports the reproducibility of VDR assessment as a clinical tool.

The increased VDR expression in polyps suggests a possible rationale for future investigation of selective vitamin D receptor modulators (VDRMs) as targeted therapy; however, this remains speculative and is not supported by functional evidence in the present study. Unlike traditional vitamin D supplementation, VDRMs could potentially provide tissue-specific effects while minimizing systemic side effects. This represents a promising avenue for developing precision medicine approaches in benign endometrial disorders [39].

The differential VDR expression patterns observed in our study have several potential therapeutic implications. Recent clinical trials in endometrial hyperplasia have demonstrated that vitamin D supplementation (50,000 IU biweekly for 12 weeks) significantly improved serum vitamin D levels and metabolic parameters [34]. These findings suggest that vitamin D pathway modulation could represent a novel therapeutic approach for managing benign endometrial proliferative conditions. Furthermore, studies in uterine leiomyomas have shown that vitamin D supplementation can reduce tumor size without significant toxicity, supporting the clinical feasibility of vitamin D-based interventions [41]. Given the overexpression of VDR in endometrial polyps demonstrated in our study, similar therapeutic approaches warrant investigation for polyp management, particularly in patients with recurrent lesions or those who are poor surgical candidates.

Future investigations should employ larger cohorts with longitudinal follow-up to assess VDR expression stability over time and correlation with clinical outcomes. Molecular studies examining vitamin D target gene expression and functional pathway activation would strengthen mechanistic understanding. Integration with metabolomic or proteomic approaches could identify novel biomarkers and therapeutic targets within the vitamin D signaling network. Given the apparent discordance between systemic vitamin D levels and local VDR expression, future studies should simultaneously evaluate both parameters to better understand their relationship in polyp pathogenesis. Additionally, functional studies examining the response of polyp tissue to vitamin D treatment could provide direct evidence of the therapeutic potential suggested by our IHC findings.

Although preliminary, these findings may have potential clinical implications. VDR expression patterns might inform risk stratification strategies for endometrial polyp management, particularly in identifying lesions requiring closer surveillance or more aggressive intervention. The results also support investigating vitamin D pathway modulation as a preventive or therapeutic approach for recurrent polyps, potentially offering a non-invasive alternative to repeated surgical procedures. Moreover, the demonstration of increased VDR expression in polyps provides a rational basis for exploring vitamin D analogs as targeted therapy. Recent advances in selective VDRMs offer the potential for tissue-specific interventions that could maximize therapeutic benefits while minimizing systemic effects [41].

The pattern of increased VDR expression observed in endometrial polyps is consistent with findings in other benign hyperplastic conditions. While our observations raise the possibility that VDR expression patterns may be relevant for biomarker research, this remains speculative. Our data should be considered as preliminary evidence requiring validation in larger, independent cohorts before any biomarker implications can be established. Additional mechanistic studies, incorporating molecular and functional assays, are needed to confirm whether this phenomenon reflects a compensatory pathway or other regulatory mechanisms.

The demonstration of increased VDR expression in endometrial polyps provides a rational biological basis for exploring vitamin D analogs and VDR-related pathways as potential therapeutic targets. However, these implications remain hypothetical and should be interpreted within the exploratory scope of this study. Larger, longitudinal investigations are required to assess the stability of VDR expression over time and its correlation with clinically relevant outcomes, including recurrence and premalignant transformation. Furthermore, future molecular studies examining vitamin D target gene expression and downstream pathway activation are necessary to improve mechanistic understanding.

Limitations

This study has some limitations that should be acknowledged. The relatively small sample size limits the statistical power and generalizability of the findings. The cross-sectional design precludes causal inference between VDR expression and the development of endometrial polyps. IHC evaluation was semi-quantitative and based on visual scoring, which, although supported by strong interobserver agreement, remains subject to potential observer bias. In addition, molecular analyses such as VDR gene expression or circulating vitamin D levels were not included, which could have provided complementary mechanistic insights. Furthermore, the study included only 17 women, and no formal sample size calculation or power analysis was undertaken. As a result, the study is underpowered to detect small-to-moderate differences, and its findings should be interpreted as exploratory and hypothesis-generating, rather than definitive.

Additionally, malignant endometrial lesions were not included as a comparison group. This choice was intentional, as the present pilot study was designed to focus on benign endometrial polyps with paired adjacent normal endometrium obtained during hysteroscopic polypectomy. In our setting, malignant cases are usually managed with hysterectomy in oncologic centers, and paired polyp/adjacent endometrium samples suitable for standardized immunohistochemistry are not consistently available. Our primary objective was to generate preliminary data on VDR expression in benign hyperplastic lesions rather than to cover the full continuum of endometrial pathology. We agree that future, adequately powered studies including hyperplasia with atypia and endometrial carcinoma are needed to clarify whether VDR expression changes progressively across normal, benign, and malignant endometrial tissue.

Finally, as this investigation was conducted at a single academic institution, the results should be interpreted with caution until validated by larger multicenter studies integrating molecular and clinical data.

## Conclusions

This study demonstrates that VDR expression is significantly increased in endometrial polyps compared to adjacent normal endometrium, with 58.8% of polyps showing positive immunostaining versus 23.5% of normal tissue. These findings raise the possibility of compensatory VDR upregulation in benign endometrial polyps. These results are exploratory and hypothesis-generating and should not be interpreted as biomarker validation. Future research should integrate molecular, hormonal, and clinical data to clarify the role of VDR in endometrial pathology.
